# Native top-down proteomics reveals EGFR–ERα signaling crosstalk in breast cancer cells dissociates NUTF2 dimers to modulate ERα signaling and cell growth

**DOI:** 10.21203/rs.3.rs-3097806/v1

**Published:** 2023-07-24

**Authors:** John Yates, Fabio Gomes, Kenneth Durbin, Kevin Schauer, Jerome Nwachukwu, Robin Russo, Jacqline Njeri, Anthony Saviola, Daniel McClatchy, Jolene Diedrich, Patrick Garrett, Alexandra Papa, Ianis Ciolacu, Neil Kelleher, Kendall Nettles

**Affiliations:** Scripps Research Institute; The Scripps Research Institute; Proteinaceous; Thermo Fisher Scientific; Wertheim UF Scripps Institute; Wertheim UF Scripps Institute; The University of Colorado Anschutz Medical Campus; The Scripps Research Institute; Scripps Research Institute; The Scripps Research Institute; Wertheim UF Scripps Institute; Wertheim UF Scripps Institute; Northwestern University; Wertheim UF Scripps Institute

## Abstract

Oligomerization of proteins and their modified forms (proteoforms) produces functional protein complexes ^1,2^. Complexoforms are complexes that consist of the same set of proteins with different proteoforms ^3^. The ability to characterize these assemblies within cells is critical to understanding the molecular mechanisms involved in disease and to designing effective drugs. An outstanding biological question is how proteoforms drive function and oligomerization of complexoforms. However, tools to define endogenous proteoform-proteoform/ligand interactions are scarce ^4^. Here, we present a native top-down proteomics (nTDP) strategy that combines size-exclusion chromatography, nano liquid-chromatography in direct infusion mode, field asymmetric ion mobility spectrometry, and multistage mass spectrometry to identify protein assemblies (≤70 kDa) in breast cancer cells and in cells that overexpress EGFR, a resistance model of estrogen receptor-α (ER-α) targeted therapies. By identifying ~104 complexoforms from 17 protein complexes, our nTDP approach revealed several molecular features of the breast cancer proteome, including EGFR-induced dissociation of nuclear transport factor 2 (NUTF2) assemblies that modulate ER activity. Our findings show that the K4 and K55 posttranslational modification sites discovered with nTDP differentially impact the effects of NUTF2 on the inhibition of the ER signaling pathway. By characterizing endogenous proteoform-proteoform/ligand interactions, we reveal the molecular diversity of complexoforms, which allows us to propose a model for ER drug discovery in the context of designing effective inhibitors to selectively bind and disrupt the actions of targeted ER complexoforms.

Nearly all critical functions in cells, including those associated with malignancies like breast tumors, are driven by proteins in complexes that often assemble via non-covalent interactions of monomeric subunits ^5^. These individual proteins exist as proteoforms due to differential splicing, sequence variations, post-translational modifications (PTMs), or mutations that can influence the formation, stability, and activity of functional protein assemblies ^6^. The ability to identify protein assemblies and to characterize their monomeric proteoform arrangements within the intracellular space provides a more accurate understanding of how biological processes are regulated by the different proteoforms that may occupy protein complexes in cells. Mutated genes in cancer cells can alter protein sequences, thus creating new proteoforms that are difficult to characterize. Here, we use the term “complexoforms” to define a protein complex formed by different monomeric proteoform arrangements ^3^. Native mass spectrometry (native MS) has emerged as a powerful complement to biophysical techniques (e.g., X-ray crystallography) for structural biology of protein complexes ^1^. Native MS provides compositional information on the architecture of intact macromolecular assemblies, whereas top-down proteomics (TDP) enables in-depth characterization of intact proteoforms ^7^. These two methods have been recently combined in a single MS method “native top-down proteomics (nTDP)” that provides detailed molecular information about protein assemblies in a single experiment ^8–11^. Despite its structural elucidation power, this MS-based approach has primarily been used on purified protein complexes, with very few applications to simple ^12,13^ or complex mixtures like cell lysates ^8,10^. This may be attributed to the notorious difficulty separating intact protein assemblies in complex mixtures, but it may also be due to a lack of bioinformatics tools that can effectively handle large-scale nTDP datasets. The low intracellular abundances of many proteoform assemblies in complex mixtures further impedes the ability to study them ^4,14^. A native FAIMS-MS^n^ strategy was developed and validated using standard and protein-metal complexes ranging from ~30 to 70 kDa (**Figures S1–5**) before identifying unique protein complexes from treatment responsive and treatment resistant breast cancer cells.

## MCF-7 and MCF-7-EGFR analysis

Following the analysis of a standard protein complex mixture, whole cell lysates of MCF-7 and MCF-7-EGFR were fractionated with native size exclusion chromatography (nSEC). EGFR amplification is clinically associated with tamoxifen resistance^15^. The effects of amplification can be reproduced in the EGFR-overexpressing cells, which grow 25% faster than the parental MCF-7 cells (**Figure S6A–B**) ^16^. SEC has been previously used under denaturing conditions for fractionation of intact proteins ^17^. As shown in **Figures S5A-B**, a nSEC separation strategy was developed using protein standards (BSA, ~66kDa; OV, ~44kDa; M, ~17kDa) and a chromatographic column with a small pore size (300 Å) to facilitate the separation of medium and low molecular weight protein complexes. The separation window and signal intensity of the nSEC method were found to be highly reproducible, which allowed enrichment of extremely low abundant complexes from breast tumor cells (**Figure S5A-B**). nSEC used a mobile phase compatible with native MS; thus, the fractions were ready for direct ESI infusion FAIMS-MS^n^ analysis to maintain the integrity of protein assemblies. After concentration of each fraction with molecular weight cutoff filters, Native-PAGE was used to visualize the nSEC fractions from each sample to confirm the presence of protein complexes (**Figures S7A-B**). These results show the complexity and composition of the two samples are similar and that protein complexes remain intact. Each nSEC fraction was then ionized by electrospray and fractionated using specific voltage settings on the FAIMS. Ions were isolated by the quadrupole mass-filter and dissociated via higher-energy collisional dissociation (HCD). Ion m/z values were recorded in the Orbitrap. Five-fractions of MCF-7 and 5 fractions of MCF-7-EGFR cells with identical retention times were analyzed, and the overall trends were preserved across triplicate analyses. A total of 104 complexoforms from 17 protein complexes were identified with high confidence. By matching theoretical and observed masses within ±1 Da, ~13 protein assemblies were identified with confidence. We also considered 3 protein assemblies (formed by truncated proteoforms) identified based only on their observed masses. A complete list of identified protein complexes/complexoforms and their proteins/proteoforms is provided in **Table S1**. We characterized three distinct groups of protein assemblies: 1) protein-metal assemblies, 2) protein-protein-metal assemblies, and 3) protein-protein assemblies (**Table S1**). The components of these protein assemblies were identified, and the stoichiometry of the assemblies defined, which is critically important to advancing our understanding of mechanism in the development of breast tumor development, progression, and drug resistance. Complexoforms were also identified containing truncated proteoforms, an irreversible PTM that can significantly affect the function and fate of protein complexes ^18^.

**Figure 1:**
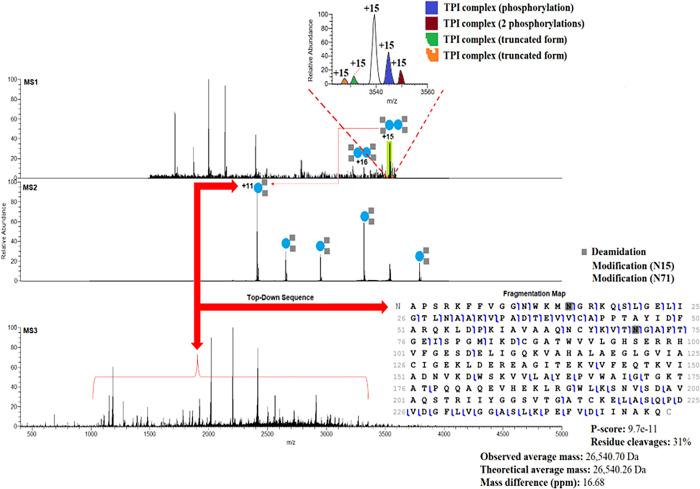
nTDP spectra of TPI complexoforms (dimeric structures) in MCF-7 cells and the fragmentation map with 2 deamidations.

Heteromeric and homomeric assemblies that ranged from dimers to hexamers were identified (**Table S1**). Novel heterodimeric assemblies of triosephosphate isomerase (TPI), heterotrimeric assemblies of macrophage migration inhibitory factor (MIF), homodimeric assemblies of superoxide dismutase [Cu-Zn] (SOD1), and heterodimeric assemblies of nuclear transport factor 2 (NUFT2) were also identified (**Table S1**). The characterization of four protein complexes in MCF-7 cells including three protein-protein and one protein-protein-metal assemblies serve as examples of our methodology’s efficiency. We initially interrogated the dimeric structure of TPI, an important glycolytic enzyme involved in numerous cancers, including breast cancer. The function of TPI in tumors is poorly understood, but studies suggest that it may be associated with drug resistance, tumor progression, and metastasis ^19,20^. In this study, we observed dimeric structures of TPI with molecular masses of ~53kDa in both MCF-7 and MCF-7-EGFR cells. Figure 1 shows the TPI dimers at the m/z range of 3318 – 3539 with two charge states (16+ and 15+). Closer examination of the MS^1^ spectrum revealed that dimeric TPI complexes are formed by truncated and phosphorylated monomeric proteoform arrangements (Figure 1). Following the release from dimeric TPI, monomeric proteoforms (11+ charge state) were isolated using the quadrupole mass filter and fragmented by HCD (Figure 1). HCD fragmentation revealed a monomeric proteoform with two deamidations (N15 and N71). The MS^3^ spectrum yielded isotopically resolved sequence ions that were mapped to the sequence of TPI and displayed in a graphical fragmentation map (Figure 1). TPI has been used to study biological catalysis; however, it has been suggested that endogenous proteolysis and deamidations of this enzyme can negatively affect its catalytic activity ^21^. Although these deamidations and their location have been previously reported ^22^, top-down analysis provides definitive confirmation of the location of these modifications. The HCD fragmentation resulted in backbone cleavages that yield diagnostic ions that allowed us to unambiguously pinpoint the deamidation sites and confirm previous observations ^23^. This high level of characterization makes it possible to define the specific proteoform associated with a particular pathological state. In Figure 1 a fragmentation map shows the sequence coverage of the deamidated monomeric proteoforms that form the TPI dimeric structure. We found TPI complexoforms that were formed by mutated (E104D) forms of TPI (**Table S1**), and we also found that E104D monomeric subunits of TPI can be phosphorylated, deamidated, or acetylated (**Table S1**). Although mutations in E104D can induce endogenous proteolysis due to loss of rigidity of the 3-dimensional structure of TPI, the biological implications of PTMs on mutated TPI remains to be elucidated. In humans, TPI deficiency has been associated with neurological diseases, cardiomyopathy, and mutations in E104D result in premature death ^24^.

**Figure 2:**
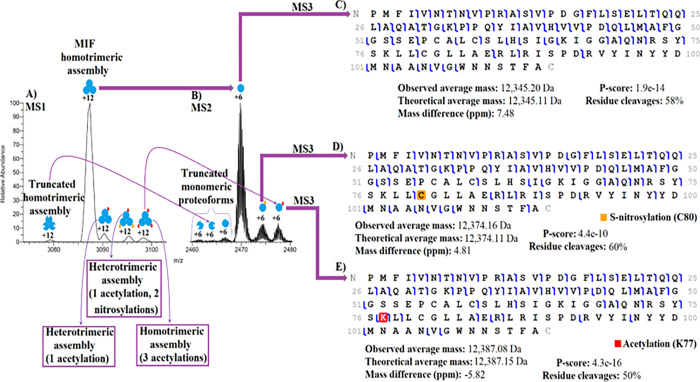
nTDP spectra of the MIF complexoforms (trimeric structures) in MCF-7 cells and its fragmentation maps (unmodified map, nitrosylated map, and acetylated map).

We found that MIF, a versatile cytokine with biological relevance in several cancers, autoimmune diseases, and inflammation ^25,26^, is highly expressed in both MCF-7 and MCF-7-EGFR cells. As illustrated in Figure 2A, the native MS spectrum provided a complete overview of MIF complexoforms. We used a wide quadrupole isolation window to isolate and fragment multiple peaks and characterized the peaks via diagnostic product ions of the constitutive proteins, which enabled the characterization of proteoforms and determination of how they were arranged in the trimeric assemblies. For instance, as shown in Figure 2A, we isolated the 12+ charge states found in the m/z range ~3080 – 3100 from MCF-7 cells, which corresponds to the trimeric structures (~37 kDa) of MIF and confirms that MIF’s native-like assemblies are retained during the Orbitrap measurements. Figure 2B illustrates the MS^2^ spectra of the monomers (6+ charge state) ejected from the 12+ charge states of the MIF trimmers using HCD activation and their respective fragmentation maps along with sequence coverage, identification confidence scores (P-Score), and mass accuracy (Figures 2C–E). When trimeric structures were disassembled, truncated, unmodified, nitrosylated, and acetylated monomeric proteoforms were revealed (Figures 2B–E). We also identified MIF complexoforms that were formed by interactions of phosphorylated monomeric subunits (**Table S1**). As shown in Figure 2A, five MIF (homo and hetero) trimeric structures were formed by different monomeric MIF proteoform arrangements. MIF truncated forms not related to MIF assemblies were also observed (Figure 2B). The truncated monomeric proteoforms were not characterized, but the three monomeric proteoforms that form four different trimeric structures are shown in Figures 2C–E. As illustrated in Figures 2D–E, the high sequence coverage accomplishedon either side of the modified residues with HCD allowed us to unequivocally assign C80 and K77 as nitrosylated and acetylated residues, respectively. It is widely accepted that S-nitrosylation can alter the biological activity and properties of many proteins; however, the biological implications of this covalent attachment on the thiol side chains of MIF’s cystines in breast cancer cells are unknown. Although S-nitrosylation at C81 has been previously reported ^27^, top-down analysis provides clear evidence of the presence and location of this PTM and its association with a protein complex.

**Figure 3:**
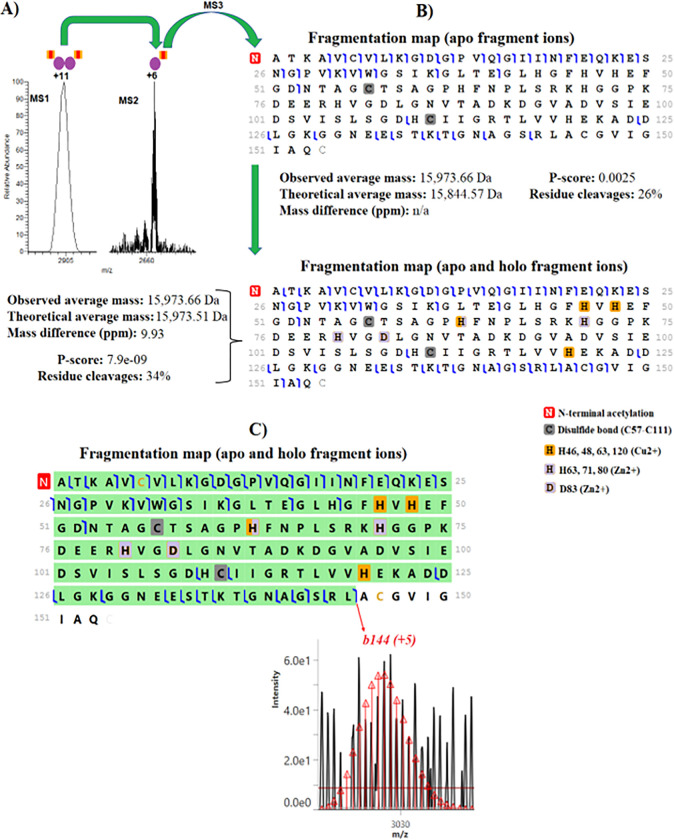
nTDP spectra of the complexoform SOD1 (homodimeric structure) in MCF-7 cells. **(A)** MS1 and MS2 spectra of SOD1. **(B)** Fragmentation maps of SOD1 with apo and holo/apo fragment ions that were generated using the ProSight Lite software. **(C)** Fragmentation map of SOD1 monomeric proteoform covering regions of the protein where the metal ions are bound and isotopic distribution of the diagnostic ion *b144* that were generated using TDValidator software.

We characterized the metalloenzyme SOD1, an important antioxidant that is regarded as a potential target for endocrine treatment ^28^ and is necessary for the conversion of superoxide into oxygen and hydrogen peroxide. Expansion of the MS^1^ spectrum reveals that native ESI-FAIMS of SOD1 allowed ions of the homodimeric assembly (~32 kDa) noncovalently bound to a Cu2+ and a Zn2+ to be efficiently transferred into the gas phase (Figure 3A). We observed noncovalent binding of the complex’s charge state 11+ to Cu2+ and Zn2+ ions in the native MS spectrum. The Cu2+ and Zn2+ ions were retained during HCD activation in the MS^2^ stage, while the monomeric proteoforms were disassembled from the complex, yielding a 6+ charge state that was isolated by the quadrupole and subsequently dissociated in the MS^3^ stage using HCD. This confirms that electrostatic interactions between the backbone side chains, and metal ions are maintained during HCD activations to yield binding site information (Figure 3A). Figure 3B gives the fragmentation maps of SOD1 using the ProSight Lite with apo fragment (non-metal) ions and holo fragment (metal) ions. Holo fragment ions identified with mass shift consistent with the binding sites of Cu2+ and Zn2+ ions. Notably, most of these holo fragment ions occurred on both N- and C-terminals and cover regions in the middle of the protein (Figure 3B), providing clear evidence that the metal ions are located in the middle of the SOD1 proteoform. A previous study has shown that the Cu2+ cation is bound at the residues H46, H48, H63, and H120 while the Zn2+ cation is bound at the residues H80, H71, H63, and D83, which is in agreement with our observations ^29^. The SOD1 proteoform also carries an N-terminal acetylation, initial methionine cleavage, and a disulfide bond (C57-C111) (Figure 3B). It remains to be determined if acetylation of this homodimeric assembly is biologically relevant. Holo fragment ions were manually verified using TDValidator to increase confidence in the localization of metal cofactors ^30^. TDValidator generated theoretical isotopic distributions of the detected holo fragment ions and overlayed them on the raw spectrum (Figure 3C). **Figure S8** gives a fragmentation map of SOD1 with holo fragment ions marked in blue and apo fragment ions marked in purple where backbone cleavages occurred upon ion dissociation. Notably, we also found SOD1 complexoforms that carry methylation (**Table S1**). Although our native top-down analysis provides unambiguous evidence of SOD1 proteoforms that carry a methylation and form homodimeric methylated assemblies, these proteoforms were identified with confidence level 2A, as the methyl group is identified in the protein backbone, but not localized ^31^. The importance of protein methylation has been observed in numerous cellular and physiological processes ^32^, but the biological relevance of methyl groups on the SOD1’s primary and three-dimensional structures in breast cancer cells are unknown. Methylations in SOD1 proteoforms and dimers have not been previously reported.

**Figure 4:**
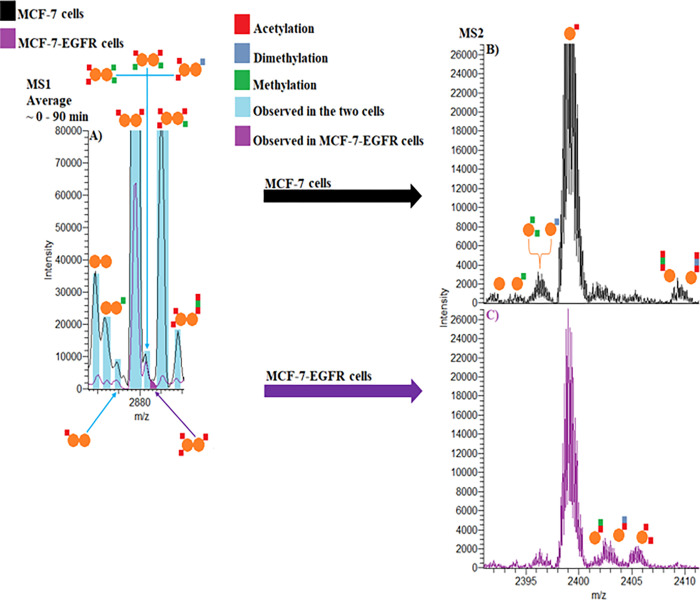
nTDP spectra of NUTF2 in MCF-7 and MCF-7-EGFR cells. **(A)** Representative native MS spectrum of NUTF2 in MCF-7 (black) and MCF-7-EGFR (purple) cells. **(B)** MS2 spectrum of NUTF2 monomeric proteoforms in MCF-7 cells. **(C)** MS2 spectrum of NUTF2 monomeric proteoforms in MCF-7-EGFR cells.

**Figure 5:**
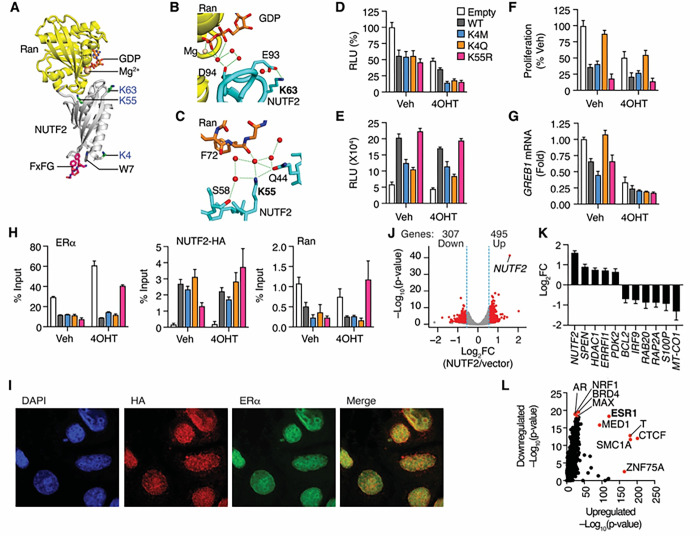
NUTF2 Modulates ERα signaling. (A–C) Crystal structure of one subunit of the NUTF2 dimer interacting with RanGDP and a model of the FxFG nucleoporin peptide, based on PDBs 1GYB and 5BXQ. (D) NUTF2 inhibits ERα-mediated transcription. 3xERE-Luc reporter activity in MCF-7 cells co-transfected with ERα, and empty or NUTF2 expression vectors, and treated in complete medium with vehicle or 1 μM 4OHT for 24 h. N = 7. (E-H) use same legend as D. (E) NUTF2 promotes DNA binding by ERα. 3xERE-Luc reporter activity in HEK293T cells co-transfected with ERα-VP16 fusion protein, and empty vector or NUTF2 expression vectors, and treated in complete medium with vehicle or 1 μM 4OHT for 24 h. N = 5. (F) NUTF2 suppresses ERα-mediated cell proliferation. Stable MCF-7 cells were cultured in complete medium supplemented with vehicle or 1 μM 4OHT for 5 days. N = 4. (G) NUTF2 downregulates *GREB1*. Stable MCF-7 cells in complete medium were treated for 24 h with vehicle or 1 μM 4OHT. *GREB1* mRNA levels were compared by qPCR. N = 2.(H) NUTF2 occupies an ERα-binding site in the *GREB1* promoter. Stable MCF-7 cells treated for 1 h with vehicle or 1 μM 4OH, were compared by qChIP assay using ERα, HA-tag and Ran antibodies. N = 3. (I) Confocal microscopy of NUTF2 expressing MCF-7 cells. Cells were fixed, permeabilized and stained with DAPI or antibodies against the HA-tagged NUTF2 plasmid or ERa. See also Figure S14. (J) Volcano plot of RNA-seq data showing the effect of NUTF2 overexpression on gene expression in MCF-7 cells, n = 3. (K) RNA-seq data showing expression profiles of select growth regulatory genes. Mean + SEM, n = 3. (L) Potential upstream regulators of NUTF2 target genes were identified based on published ChIP-seq data sets using LISA (http://www.lisa.cistrome.org).

Finally, we characterized the NUTF2 protein in the two cell lines. NUTF2 is a nuclear protein that mediates the nuclear import of Ran, a small GTPase that directs nucleocytoplasmic trafficking of cargo proteins with a nuclear localization sequence (NLS), and other proteins ^33,34^. Our nTDP approach revealed that EGFR induces the dissociation of NUTF2 dimeric assemblies. There is a dramatic change in complexoform and proteoform expression patterns in response to EGFR overexpression. We observed a substantial decrease of the endogenous NUTF2 dimeric complexoforms in MCF-7-EGFR when compared to MCF-7 cells (Figure 4A and **Table S2**). The NUTF2 heterodimer with three acetylations seemed to be unique for MCF-7-EGFR cells (Figure 4A). Studies have shown that NUTF2 levels are directly related to efficient regulation of protein transport between nucleus and cytoplasm and there is a significant portion of monomeric NUTF2 forms in the cellular environment ^35,36^. Following HCD activation for the MS^2^, we observed numerous monomeric NUTF2 proteoforms including acetylated, methylated, and dimethylated forms (Figures 4B–C). Initial methionine cleavage was observed in all NUTF2 proteoforms. We also observed differences in the expression of the monomeric NUTF2 proteoforms among the two cells, shown in bold (**Table S3**), while the overall expression was similar (**Figure S9**). Although we observed a peak potentially arising from either of two monomeric products including 1) K4 acetylated-K55 methylated-K63 acetylated form, or 2) K4 acetylated-K55 dimethylated-K63 acetylated in both cells, there was no HCD evidence (MS^3^ level) for these proteoforms and identifications were based on the mass shift of the MS^2^ spectra (Figures 5B–C, **Figures S10A-B**). Our nTDP strategy also revealed that NUTF2 interacts with SOD1 and D-dopachrome decarboxylase (DDT), forming NUTF2-SOD1-DDT, NUTF2-SOD1, and NUTF2-DDT assemblies (**Table S1** and **Figures S11A-C**). While acetylations in NUTF2 subunits are known, the methylations, dimethylations, and multiple PTMs in a single NUTF2 monomeric subunit have not been previously reported.

## NUTF2 mediates crosstalk between the EGFR and ERα signaling pathways

The NUTF2 PTM sites that we identified are conserved in vertebrates, with variable conservation in other species (**Fig S12**). Crystal structures of NUTF2 protein complexes indicate that K4 is near the nucleoporin binding site (Fig 5A). K55 and K63 are close to the Ran binding site, with K55 participating in water mediated contacts between Ran and NUTF2 (Fig 5B–C) ^37,38^, leading us to mutate K4 and K55.

To determine whether NUTF2 modulates ERα activity in MCF-7 cells, we used an estrogen-response element (ERE)-driven luciferase assay to compare the effects of NUTF2 on ERα-mediated transcription. Wildtype NUTF2 reduced ERα activity, which was further reduced by treatment with the active metabolite of tamoxifen, 4OHT, while mutation of the K4 or K55 further reduced ERα activity (Figure 5D). To determine whether NUTF2 modulates DNA binding by ERα, we examined its effect on ERE-luciferase reporter activity driven by fusion of ERα to the strong transcriptional activation domain of herpes simplex virus protein VP16 ^39^. Wildtype NUTF2 increased ERα-VP16 fusion protein-driven reporter activity (Figure 5E), indicating that NUTF2 promotes DNA binding by ERα to a canonical estrogen response element. The K4M and K4Q mutations reduced the effect (Figure 5E) and control of this activity.

We engineered MCF-7 cells to stably express epitope tagged NUTF2 proteins since MCF-7 cells were not viable after transduction with short hairpin RNAs to silence the *NUTF2* gene. Exogenous NUTF2 levels did not exceed the levels of the endogenous protein in these stable cells (**Figure S13A**), and none of the mutations affected total NUTF2 protein levels (**Figure S13B**). However, the K4Q mutation increased NUTF2 levels in the water-soluble cell fraction, and increased expression of Ran (**Figure S13C–D**). K4 acetylated NUTF2 was the predominant species detected by top-down proteomics (**Tables S2** and **S3**). Together, these findings suggest that K4 acetylation drives NUTF2 into soluble nuclear compartments or prevents its ubiquitylation.

Exogenous NUTF2 suppressed cell proliferation (Figure 5F), as seen in melanoma ^40^. The K4Q mutation rescued this phenotype (Figure 5F), suggesting that K4 acetylation blocks the growth-inhibitory activity of NUTF2. Similarly, exogenous NUTF2 downregulated the estrogen-induced proliferative gene, *GREB1*, and the K4Q mutation also rescued this phenotype in the absence of 4OHT (Figure 5G). NUTF2 was recruited to consensus ERE in the *GREB1* promoter region (Figure 5H), but not at the *ROCK2* promoter, a nearby regulatory region lacking an ERα-binding site ^41,42^ (**Figure S13E**), suggesting that NUTF2 is specifically recruited to repress ERα-mediated transcription of *GREB1*. These effects were only blocked by the K55R mutant. NUTF2 and Ran were differentially recruited to this site (Figure 5H), while the K55R mutant blocked the inhibition of Ran binding only in the presence of 4OHT. Nuclear localization of NUTF2 was not altered by the mutants (Figure 5I, **Figure S14**). These experiments demonstrate that the K4 and K55 PTM sites discovered with nTDP differentially impact unique aspects of NUTF2 effects on the ERa signaling pathway.

We completed RNA-seq of NUTF2 overexpressing versus empty vector MCF-7 cells, with more genes being upregulated (Figure 5J). Gene set enrichment analyses suggest that NUTF2 upregulated RNA catabolic processes, components of the NuRD complex such as HDAC1 and SPEN1, and transcriptional repressor complex (Figure 5K, **Figure S15A–C**). The apoptotic gene *ERFFI1* was also upregulated, as was *PDK2*, the suppressor of aerobic respiration. Genes encoding mitochondrial proteins including those that mediate oxidative phosphorylation were downregulated, such as the antiapoptotic gene, *BCL2*, and respiratory chain gene *MT-CO1* (Figure 5K, **Figure S15D–E**). The oncogenic RAS family genes *RAB20* and *RAP2A*, five PARP family genes, and several genes involved in the interferon signaling pathway (including IRF9) were also downregulated (Figure 5K, **Figure S15F–G**). We also noted that there were 51 genes that are regulated by estradiol in quiescent MCF-7 cells, highlighting the broader role of NUTF2 in regulating gene expression (**Figure S16**). This role was also supported by an analysis of ChIP-seq data sets, which identified ERa binding sites among the top five upstream regulatory elements of both upregulated and downregulated genes, but other transcription factors and coregulators were also identified (Figure 5L). Altogether, these results support a new EGFR–ERα signaling crosstalk mechanism in breast cancer cells, where EGFR signaling dissociates NUTF2 dimers and alters the PTM code to modulate ER signaling and cell growth, along with other critical drivers of cancer growth and metabolism.

## Conclusions

Efficient fractionation of low abundance protein complexes allowed us to identify the proteoforms that constitute different protein assemblies. Our platform opens a new avenue for large-scale native top-down proteomic characterization of endogenous complexoforms. It is uniquely positioned to reveal intracellular molecular pathways and illuminate functional PTM differences in the expression landscapes of complexoforms and their proteoforms. By advancing the understanding of the higher-order organization of cancer cells, this work enhances functional assignment and inspires design of cancer therapeutics.
